# Amine-Polyether-Epoxide Nanoplatform-Driven Seed Germination, Plant Growth, and Nutrient Uptake for Sustainable Agriculture

**DOI:** 10.1021/acsomega.4c11661

**Published:** 2025-03-20

**Authors:** Bruno A. Fico, Heber E. Andrada, Felipe B. Alves, Enzo E. da Silva, Julia S. Reinaldi, Denise C. Tavares, Iara S. Squarisi, Laura G. Nuevo, Gabriel Sgarbiero Montanha, Hudson W. P. de Carvalho, Fabián Vaca Chávez, Eduardo F. Molina

**Affiliations:** † Universidade de Franca, Av. Dr. Armando Salles Oliveira 201, Franca, São Paulo 14404-600, Brazil; ‡ Grupo de Estudo em Fertilizantes Especiais e Nutrição, Centro de Energia Nuclear na Agricultura, Universidade de São Paulo, Av. Centerário 303, Piracicaba, São Paulo 13400-970, Brazil; § Facultad de Matemática, Astronomía, Física y Computación (FAMAF), Universidad Nacional de Córdoba, Córdoba X5000, Argentina

## Abstract

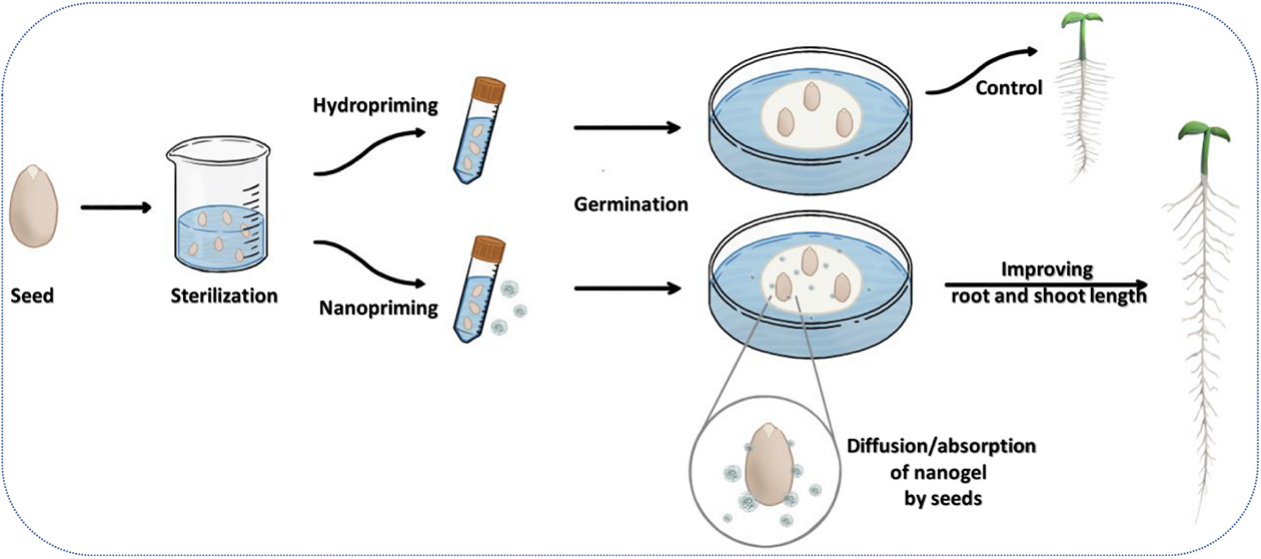

Polymeric systems can facilitate the diffusion of micronutrients through seeds, offering an innovative and sustainable way to improve plant health and increase food production. In the present work, a polymeric nanogel based on polyether-POE-diamine and bisepoxide was synthesized and in-depth characterized, encompassing its morphological characteristics (by Transmission Electron Microscopy, TEM), the size distribution, and surface charge of the particles (by dynamic light scattering, DLS and zeta potential, ζ). The formation of the polymeric network was assessed using Fourier-transform infrared spectroscopy (FTIR) and proton nuclear magnetic resonance (^1^H NMR), confirming the opening of the epoxide ring and the formation of amine and glycol groups. The effects of seed priming with the nanogel (well-defined spherical particles, with sizes around 120 nm, named POE-gel) on the early growth stage of cucumber plants (*Cucumis sativus*) exhibited a discernible ameliorative impact on root and shoot lengths, with average improvements of 33% and 90%, respectively, compared to the control group after 12 days. Extensive investigation using germination assays and micro X-ray fluorescence (μ-XRF) analysis indicated potential applications of the POE-gel as a carrier for micronutrients (such as Fe^3+^). The seeds treated with the iron-loaded POE-gel presented a substantial positive effect on root length, exhibiting a 3-fold increase in size compared with the control-Fe treatment at the same concentration. The loaded POE-gel effectively penetrated through the seed compartments, providing an even distribution of iron ions and facilitating the uptake of nutrients (K, Mn, and Zn) by the seeds. Toxicological assays using zebrafish (*Danio rerio*), seeds, and leaves revealed notable safety of the iron-loaded and unloaded POE-gel for agricultural purposes. Employing water as the only solvent in the synthesis, as well as eliminating the use of a catalyst, makes this class of polymeric particles suitable for sustainable agricultural applications. The findings of this work contribute to the development of sustainable agriculture, presenting an innovative approach to enhancing plant development and nutrient uptake through the application of polymeric nanogels as a seed priming technology.

## Introduction

The global population is estimated to reach 10 billion people by 2030,^1^ requiring concerted efforts to ensure the sustainability of crop cultivation and achieve high levels of productivity.^2^ Over the years, in both academia and industry, there has been a notable increase in interest in the development and application of polymer-based materials within the agricultural sector.^3−5^ The distinctive physicochemical properties of polymers confer versatility for use in traditional applications and, more recently, in the areas of health, nutrition, and environmental pollution control.^6,7^ The strategic design and implementation of smart polymers are creating new opportunities for the improvement of operational efficiency in both agricultural and industrial food production processes.^8^

The use of polymers in agriculture can provide benefits, including the optimization of crop production and the controlled release of agrochemicals.^9−12^ In the food industry, they can enhance production processes, as well as be used as additives^13^ or in improved food packaging technologies.^14^ Reports provide evidence that the use of polymers in both micro- and nanoformulations of fungicides and chemical pesticides can offer enhanced properties compared to conventional methods.^15−17^ Furthermore, the size,^18^ hydrophobic/hydrophilic balance,^19^ degree of cross-linking,^20^ and surface porosity of polymer systems^21^ can influence the loading capacity for agrochemicals within the polymeric particles, as well as the subsequent release kinetics. The use of polymers can have a positive influence on various aspects of plant development, including germination, growth, evapotranspiration, flowering, and fruit formation.^22,23^

An interesting approach involving polymers that has attracted increasing attention is the utilization of aqueous dispersions containing micro- or nanoscale hydrogel particles within physically or chemically cross-linked polymer networks, known as nanogels or microgels.^24−26^ The synthesis and applications of such colloidal polymer networks were first comprehensively reviewed in the literature in 2002.^27^ They have shown promising potential as carriers for the delivery of biomacromolecules and drugs,^28,29^ as well as serving as imaging agents.^30^ More recently, hydrogel particles have been investigated for topical drug delivery,^31^ as smart temperature- and pH-responsive gels,^32,33^ and as bioactive dental implant coatings to prevent infection.^34^ However, there is still a scarcity of information in the literature concerning the exploration of polymeric micro- and nanogels for agricultural purposes.

Establishing links among polymer science, nanotechnology, and sustainability is essential for optimizing technologies in the agricultural sector, as reported in recent studies.^35−37^ The use of combinations of different nanoparticles or employing hybrid approaches (such as polymer–metal systems) can enhance the transport and uptake of nutrients in plants, although there is still a lack of agriculture-focused investigations concerning these multifunctional materials.^38^ Reported challenges associated with the employment of these innovative formulations include the development of a multifaceted process involving multiple steps for obtaining nanoparticles; the use of both physical (grinding/milling) and chemical (sol–gel, precipitation) methodologies for nanoparticle preparation; the implementation of environmental safety protocols; and ensuring cost-effectiveness and expeditious mass production after scale-up.^39^

This work contributes significantly to addressing these issues by demonstrating the attractive properties of a polymeric nanogel. Recently, our group used branched poly(propylene oxide) diamine PPO-chains to synthesize polymeric microgels for agricultural applications.^40^ In contrast to previous work,^40^ the present study employs a reaction between linear polyetheramine based on polyoxyethylene diamine POE-chains and an epoxy monomer, resulting in the formation of smaller spherical particles (nanogels). Notable features of this POE nanogel include the utilization of water as the only solvent, with no need for a catalyst in the reaction; an engineered interior exhibiting networks containing amine, alcohol, and ether-type oxygen groups for loading various micronutrients and specific metal ions; and the capability for functionalization/modification. This research primarily aimed to evaluate the potential of POE nanogels as seed priming technology. Specifically, the objective was to assess the impact (positive or negative) of their application to seeds during the early stages of plant development. The observed positive effects prompted the incorporation of an iron-based micronutrient, allowing us to further demonstrate the efficiency of this polymeric formulation as a nutrient carrier within the seed compartments. Furthermore, novel insights about the influence of metallomic distribution of elements such as iron (Fe), potassium (K), calcium (Ca), manganese (Mn), and zinc (Zn) within cross-sections of seed coats, embryos, and cotyledons of *C. sativus* seeds primed with a control Fe solution and Fe-loaded POE nanogel were investigated.

## Materials and Methods

### Chemicals

Diepoxy poly(ethylene glycol) (DPEG, C_3_H_5_O_2_-(C_2_H_4_O)_*n*_-C_3_H_5_O, *M*_W_ = 500 g·mol^–1^; CAS 26403-72-5) was purchased from Sigma-Aldrich. Jeffamine polyetheramine ED-2003, containing polyoxyethylene chains (POE, *M*_W_ ∼ 2000 g·mol^–1^) was donated by Huntsman Chemical. Ethylenediaminetetraacetic acid iron(III) sodium salt (Fe-EDTA) was purchased from Sigma-Aldrich. All of the reagents were used as received.

**Scheme 1:**
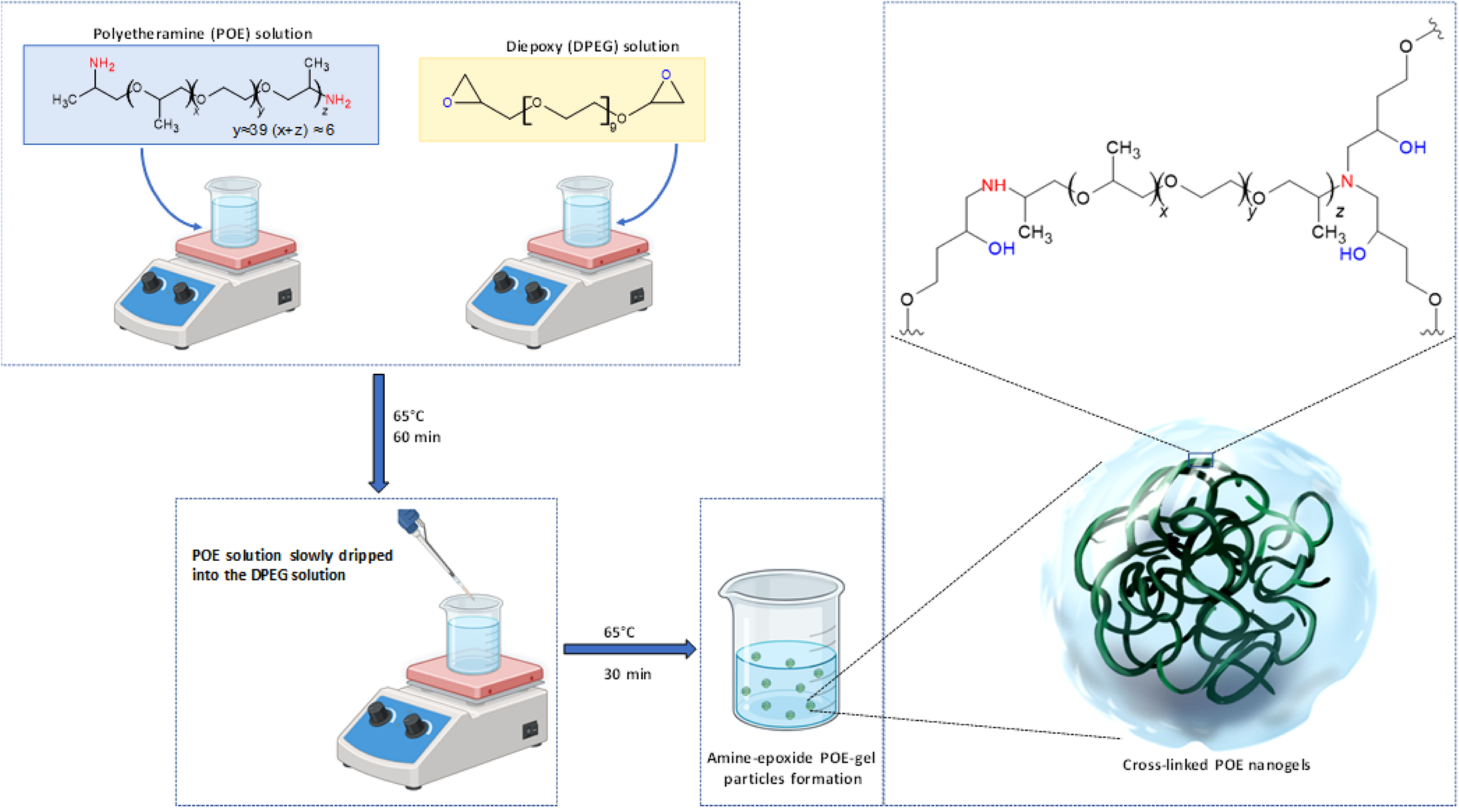
Representation of the Process for Formation of the POE-Gel Based on Polyetheramine (Jeffamine ED-2003) and DPEG (Epoxide) in an Aqueous Environment

Amine-polyether-epoxide synthesis and characterization: In the initial step, the monomers were solubilized in ultrapure water for 60 min at 65 °C, with continuous stirring. Specifically, DPEG and POE (Jeffamine polyetheramine) were individually dissolved in 5 mL of ultrapure water to achieve a total monomer concentration of 10 wt %, with a POE:DPEG molar ratio of 1:1. After stirring for 60 min, the polyetheramine solution was gradually introduced dropwise into the DPEG solution, and the resulting monomer mixture was heated in a water bath for 30 min at 65 °C, followed by cooling to room temperature.^41,42^ The amine-epoxide mixture was then diluted to 0.5 wt % with ultrapure water, obtaining the final polymeric nanogel, denoted as POE gel. This POE-gel formulation (at 0.5 wt %) was used in all the seed priming assays. It is well established that the reaction of the amino-terminated groups (from the linear or branched polyether backbone) and epoxide produces robust nanogels with a cross-linked structure, where −NH and −OH groups are dispersed throughout the three-dimensional network.^43,44^Scheme 1 shows the formation of the amine-epoxide POE-gel, with the presence of ether-type oxygen, amine, and glycol groups. After the initial ring opening by an amino-terminated group, the structure formed with −NH could react with another epoxide, leading to the polymeric structure with the ability to cross-link, resulting in the formation of a structured polymeric network.

The hydrodynamic diameter (*D*_h_), polydispersity index (PDI), and zeta potential (ζ) of the POE-gel were evaluated using a ZSU3100 Zetasizer Lab Blue instrument (Malvern Panalytical) equipped with an OBIS solid-state laser source (λ = 633 nm). The experiments were performed at room temperature (25 °C) and were repeated three times independently. The data were expressed as mean ± standard deviation (SD) (*n* = 3). The surface characteristics of the POE-gels were investigated by transmission electron microscopy (TEM) using a JEM 100CXII instrument (JEOL) operating at 100 kV. For the analysis, a drop of the nanogel solution was deposited onto a copper grid and allowed to dry at room temperature for 1 h before the measurement. ^1^H NMR measurements were performed for aqueous solutions of the two monomers (polyetheramine-POE and DPEG) and the nanogel synthesis reaction products obtained from their combination using a Spinsolve 80 Ultra instrument (Magritek). This is one of the most powerful tools currently available for elucidating molecular structures, particularly organic molecules in aqueous solution. Confirmation of the formation of the POE-gel employed a Frontier spectrometer (PerkinElmer) equipped with an attenuated total reflection (ATR) accessory. The spectra were collected in the range of 4000–700 cm^–1^, with an average of 30 scans at a maximum resolution of 2 cm^–1^.

Seed priming and germination assays: Cucumber (*C. sativus*) is one of the most widely cultivated and consumed vegetable crops globally. It is frequently used as a model species in agricultural research and ranks among the top four most extensively grown vegetables worldwide, alongside tomatoes, cabbages, and onions.^45^ Cucumber seeds were subjected to a surface sterilization process involving sequential 15-min washes in 2% sodium hypochlorite, followed by three rinses in deionized water. The seeds were primed with the nanogel to investigate its effect on germination and shoot and root development.^46^ For this, 15 seeds were immersed in a flask containing 35 mL of the polymeric POE-gel solution, followed by incubation for 24 h in the dark at room temperature, before germination on Petri dishes. A control group with the seeds immersed in water (without a polymeric gel) was established for comparison. The seeds coated with the POE-gel, as well as control seeds, were placed on filter paper in Petri dishes with three seeds per dish (a total of five dishes for each group). The dishes were sealed with Parafilm and enclosed in plastic bags to minimize water loss. The same procedure was followed in the seed assays with iron ions (Fe^3+^). Iron(III)–sodium ethylenediaminetetraacetate solutions (concentrations of 10, 50, and 100 mg·L^–1^) were used as controls for comparison with another batch employing POE-gel with embedded Fe. The embedded POE gel was prepared using 50 mg of POE gel with 10 mL of Fe^3+^ solution (10, 50, and 100 mg·L^–1^), followed by lyophilization to ensure the incorporation of Fe into the polymeric structure and rehydration with ultrapure water. All the experimental values are reported as mean ± SD. GraphPad Prism v10.1.1 (GraphPad Software Inc., USA) and OriginPro v9.0.0 were used for statistical analyses. A one-way analysis of variance (ANOVA), followed by Tukey’s comparison test, was employed to compare the means and determine significance.

Micro X-ray fluorescence (μ-XRF) seed analysis: Microchemical imaging by μ-XRF was used, as described previously,^47^ to investigate the distributions of Fe^3+^ and other simultaneously detected analytes (K^+^, Ca^2+^, Mn^2+^, and Zn^2+^) in the tissues (seed coat, embryo, and cotyledon) of cucumber seeds primed with either Fe or POE-gel solutions (as described above). Briefly, the treated *C. sativus* seeds were medially cross-sectioned with a scalpel, preserving the embryo, and then mounted within two polypropylene thin film foils (FPPP25-R3, VHG) fixed on X-ray sample cups (Chemplex no. 1530). The analyses employed a benchtop microprobe XRF system (Orbis PC, EDAX, USA) equipped with an Rh anode, operating at 45 kV and 500 μA, with focusing by polycapillary optics to obtain a 30 μm beam. The distributions of Fe^3+^, K^+^, Ca^2+^, Mn^2+^, and Zn^2+^ in the seed tissues were assessed either by 64-point line-scanning or by 800-pixel matrix mapping, as detailed in Figure S1. The spectra were recorded by a 30 mm^2^ silicon drift detector (SDD), with 15 s·point^–1^ and 1 s·pixel^–1^ for the line-scans and maps, respectively, and the dead time was <5%. The XRF line-scans were performed using three independent biological replicates, while the maps were recorded using one replicate. The recorded elemental signals that were above the instrumental limits of detection (calculated as described elsewhere^47^ were considered valid and were normalized using the corresponding Rh Kαintensities. Finally, the line-scan elemental intensities for each seed tissue, namely seed coat, cotyledon, embryonal axis, and plumule, as shown in Figures S2 and S3, were selected and compared across the treatments using the Mann–Whitney test at a 95% confidence level (*p* < 0.05). All the analyses were performed using GraphPad Prism v10.1.1 (GraphPad Software Inc., USA) and Fiji v2.1.0/1.53c open-source software.

### Zebrafish Acute Toxicity Assays

The mineral water used in the experiments with zebrafish (*D. rerio*) had the following physicochemical characteristics: a temperature of 26 °C, a pH of 7.2, a dissolved oxygen concentration of 80 mg·L^–1^, and a conductivity of 65 μM·cm^–1^ (as averages for measurements performed throughout the experimental period). Adult zebrafish (six months old), with a body weight of 0.36 ± 0.09 g and a length of 3.60 ± 0.26 cm, were obtained from a local commercial source. The fish were acclimated in aquaria containing mineral water, under aeration, for 14 days prior to the start of the assays.^48^ For the experimental exposures, a static system was employed, using POE-gel concentrations of 50, 75, 100, and 1000 mg·L^–1^, with seven fish per group. The assays included a negative control group (water) and a reference control group (standardized propolis extract at 25 mg·L^–1^). The use of the standardized propolis extract was based on its potential toxicity at the concentration employed (25 mg·L^–1^) and enabled an assessment of the sensitivity and reproducibility of the experimental batch. During the 96-h exposure to the POE-gel, observations were made at 24, 48, 72, and 96 h. The parameters assessed included mortality and visible abnormalities related to equilibrium (loss of balance, unusual head positioning, floating, or sinking) and swimming behavior. All the experiments involving adult zebrafish complied with the guidelines of the Organization for Economic Cooperation and Development.^49^ The experimental protocols were subjected to ethical evaluation and received approval from the Ethics Committee on the Use of Animals at the University of Franca (CEUA n° 2985080121). The experiments were conducted using both male and female adult zebrafish.

Following the observation period, the animals were euthanized using benzocaine diluted (1:20,000) in 98% ethyl alcohol (0.1 g·mL^–1^). The surviving fish population was employed for the assessment of the potential genotoxicity of the POE-gel using the micronucleus (MN) test with peripheral blood, according to the protocol described elsewhere.^50^ Briefly, a small blood sample was obtained by caudal puncture, immediately spread on clean glass slides, air-dried, fixed in absolute methanol for 20 min, and subsequently stained with 10% Giemsa for 10 min. Two slides were prepared per fish. The evaluation of micronuclei (MNi) frequency in the erythrocytes involved scoring 5000 intact cells per fish at 1000× magnification. Micronuclei were identified by their distinct morphological characteristics: spherical or ovoid extranuclear bodies in the cytoplasm, a diameter of 1/3–1/20 of the main nucleus, nonrefractory nature, similar texture to the main nucleus, and complete separation from the main nucleus.^51^ A total of 12 animals were included in the micronucleus assay for a given sample.

The frequency of micronuclei (% MNi) was calculated as follows:^52^
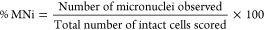


This formula provides a quantitative measure of the genotoxic impact, enabling a comprehensive evaluation of potential adverse effects on the zebrafish population induced by the POE-gel.

## Results and Discussion

**Figure 1:**
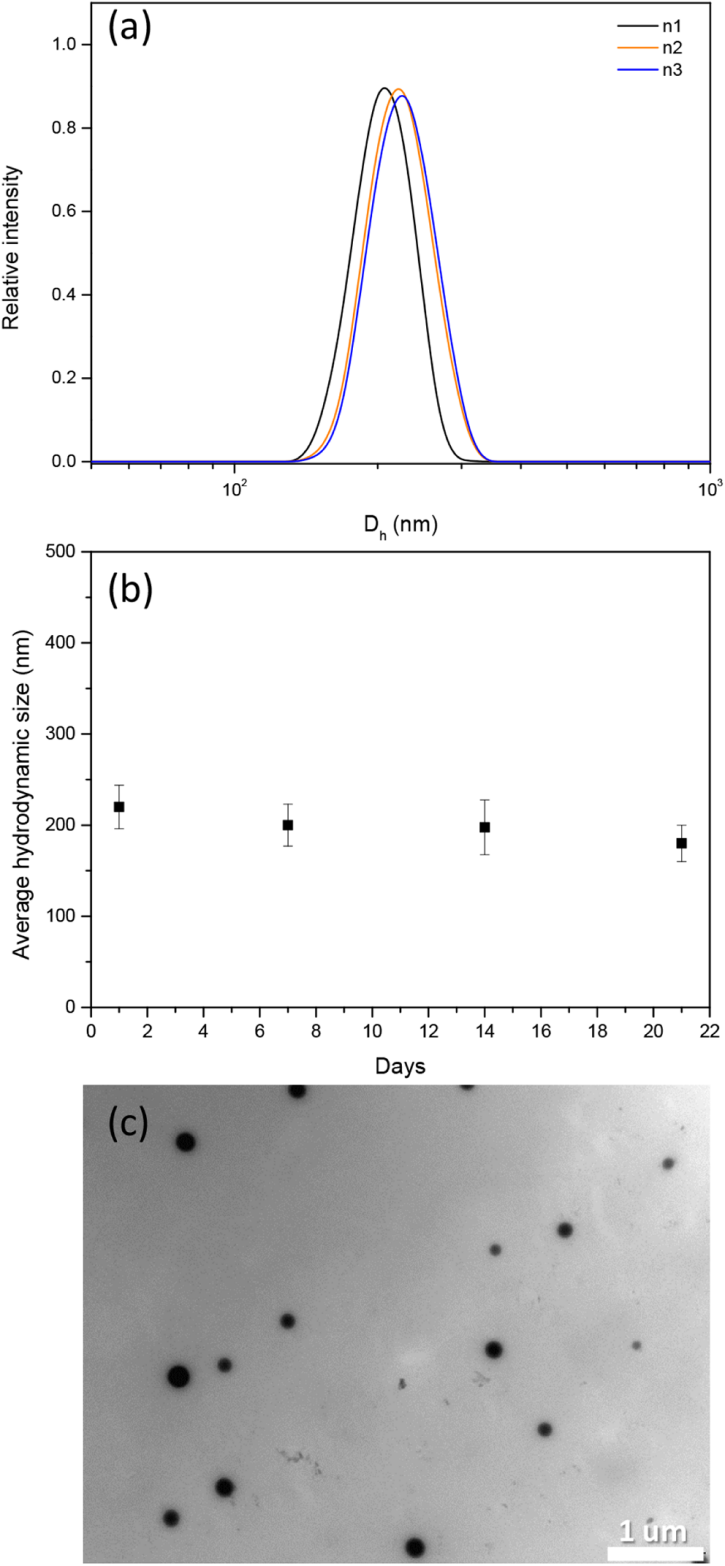
a) Hydrodynamic diameter (*D*_h_) distribution plot for the POE-gel, obtained by DLS analysis (*n* = 3). b) Hydrodynamic diameter of the POE-gel, as a function of time (mean ± SD, *n* = 3). c) TEM image of the POE-gel. The white bar indicates 1 μm.

To confirm the formation of the polymeric particles, physicochemical features characterizing the POE-gel were determined, including the hydrodynamic size distribution, polydispersity index (PdI), zeta potential (ζ), temporal stability, and morphology of the particles resulting from the reaction between poly(etheramine)-POE and diepoxide DPEG (Figure 1). The POE-gel presented an average particle diameter *D*_h_ of 220 ± 20 nm (DLS mean ± SD, *n* = 3, Figure 1a) and a polydispersity index (PdI) of ∼0.45. This PdI value for the solution could be explained by the fact that the nanogels attained different sizes, as smaller polymeric particles tended to aggregate to become more stable in aqueous solution, resulting in a variable size distribution of the nanogels produced. The ζ-value for the POE-gel was −21 mV (±1 mV) at pH 6, indicating that the nanogels were relatively stable in solution, as they reached their equilibrium size, at which they were hydrated and stabilized by the solvent (water) through hydrogen bonding. The negative zeta potential suggested the presence of negatively charged groups, which could be −OH (glycol groups) that had become deprotonated.

To investigate the temporal evolution (stability) of the POE-gel, *D*_h_ was monitored at 7-day intervals up to the 21st day (Figure 1b), with the *D*_h_ values consistently in the range of 180 to 220 nm. The TEM micrographs showed well-defined spherical nanogel particles, with sizes ranging from approximately 120 to 220 nm, formed by the aggregation of smaller polymeric particles (Figure 1c). Given the nanometric dimensions and evident stability of the polymeric system, the POE-gel was employed as a priming agent to improve seed germination, with its use in agricultural applications in mind. To elucidate its efficacy, cucumber seeds were selected as models for use in germination assays.

The ^1^H NMR spectra were used to characterize the monomers (polyetheramine-POE and DPEG epoxide) and the polymeric POE-gel network (Figure S4). The signals from the terminal methyl groups (−CH_3_) could be seen at around δ = 1.0–1.2 ppm, while the methyl groups in the chain showed signals at δ = 1.3–1.4 ppm. The methine protons (−CH−) adjacent to the amine groups showed signals at δ = 2.6–2.9 ppm, attributed to deshielding effects caused by the nearby amine groups (−NH_2_) (Figure S4a). Additionally, peaks related to the methylene protons in the polyoxypropylene backbone (−CH_2_−) could be seen near δ = 3.4–3.6 ppm, corresponding to the ether groups (−O−) present along the main chain. The DPEG spectrum (Figure S4b) showed peaks at around δ = 3.2–4.0 ppm, corresponding to the methylene protons in the polyethylene glycol backbone (−CH_2_–O−), reflecting the (−CH_2_−) groups adjacent to the ether oxygen atoms. Signals in the range δ = 2.6–2.8 ppm corresponded to the methylene protons of the epoxy ring (−CH_2_–O−), crucial for tracking the ring-opening reaction. Protons adjacent to oxygen in the epoxy ring (−CH–O−) showed signals at around δ = 2.8–3.0 ppm, due to their proximity to the oxygen atom within the ring.^53−55^ In the spectrum for the reaction between polyetheramine-POE and DPEG diepoxide (Figure S4c), a significant reduction in signal intensity for the epoxy ring protons (δ = 2.6–2.8 ppm) confirmed the ring-opening by reaction with amine groups (from the polyetheramine reagent), resulting in the formation of new covalent bonds with the amine groups. The signal for the protons belonging to these methylene groups, which reacted and formed chemical bonds with the amine groups, was present at a higher chemical shift of around δ = 3.0 ppm. Hydroxyl groups (−OH) were also generated during this process. Signals for the hydroxyl protons were difficult to observe due to rapid relaxation and exchange with water, although a small peak was detectable at around δ = 4.6–4.8 ppm, a region where amine (−NH−) protons may present signals.^56^

FTIR measurements were performed to further investigate the formation of the amine-epoxide POE-gel network. The FTIR spectrum of polyetheramine-POE (Figure S5, black line) presented a band at around 2870 cm^–1^, attributed to stretching vibrations of the amine groups (−NH_2_). Superimposed on this band were the stretching vibrations of the methyl (−CH_3_) and methylene (−CH_2_−) groups from the polyoxypropylene backbone. Additionally, bands in the regions 1465–1480 cm^–1^ and 1000–1150 cm^–1^ could be attributed to the bending vibrations of the methyl groups and the stretching vibrations of the ether groups (C–O–C) in the POE chains, respectively. The spectrum for DPEG (Figure S5, red line) featured a characteristic band corresponding to epoxide groups, centered at 910 cm^–1^, which was crucial for monitoring the reaction since the intensity of this band decreased when epoxide ring-opening occurred. The C–H stretching vibrations of methylene groups (−CH_2_−) from the polyethylene glycol chain were visible at around 2870 cm^–1^, together with ether (C–O–C) vibrations at around 1000–1150 cm^–1^.^57,58^

The green line in Figure S5 shows the spectrum for the reaction between poly(etheramine)-POE and DPEG for nanogel formation. The absence of the band centered at 910 cm^–1^, characteristic of epoxide groups, was due to the successful opening of the epoxide rings since they reacted with the amine-terminated groups of POE to form the polymeric network. Additionally, an increase of the band at around 3200–3500 cm^–1^ could be attributed to the formation of new hydroxyl groups (−OH).^59^ Therefore, the DLS, TEM, NMR, and FTIR analyses were consistent in evidencing the formation of the POE-based nanogel.

**Figure 2:**
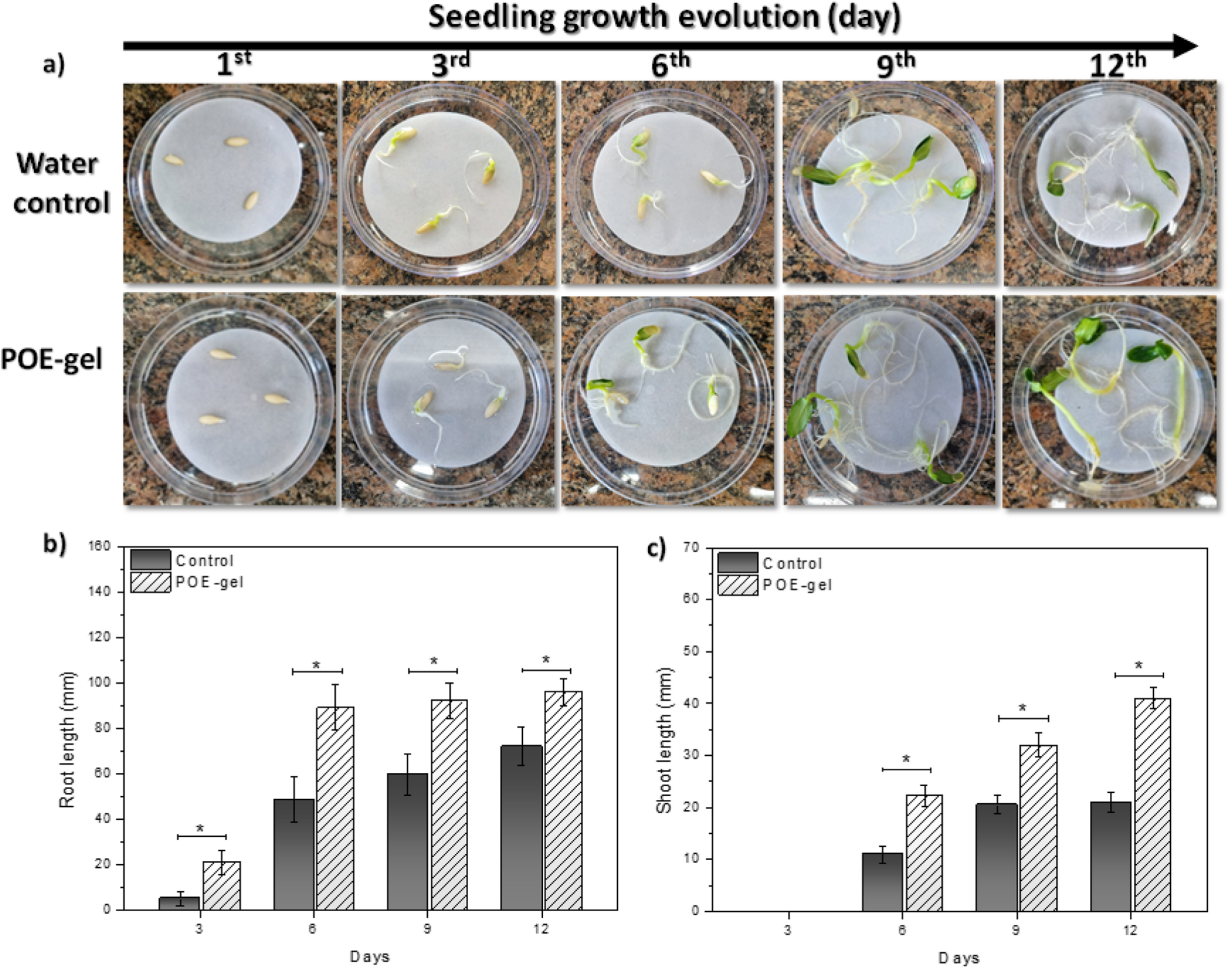
Cucumber seed germination progress over a 12-day period. (a) photograph of the seeds evolution by comparing use of the synthesized POE-gel and the water control. (b) the root and (c) shoot lengths of *C. sativus* seedlings measured at 3, 6, 9, and 12 days of growth after hydropriming (water as control) and POE-gel (nanoprimimg). Statistical significance was determined using one-way ANOVA with multiple comparisons (*p < 0.05).

Figure 2 illustrates the germination and development of the seeds over a 12-day period. Following the seed priming treatment, the seeds were arranged in Petri dishes and germinated in ultrapure water. For comparison, ultrapure water was employed as the control medium during the seed priming process, commonly referred to as hydropriming. The use of the POE-gel led to a notable enhancement of germination (Figure 2a), as evidenced by the faster emergence of the treated seeds, starting from the first day, compared to the control seeds. This favorable trend persisted throughout the experimental period, culminating in significant root and shoot development by the 12th day. These preliminary findings indicate the potential utility of the POE-gel as an aqueous system employing a nanopriming agent, capable of enhancing the germination processes of cucumber plants without inducing adverse effects.

Figure 2b,c shows the root and shoot lengths of cucumber seeds subjected to priming with the POE-gel or with water as the control aqueous medium. The results demonstrated that the priming treatment with the POE-gel was effective in improving seedling growth compared to the seeds exposed to the water-control. The use of the POE-gel exhibited an ameliorative impact on the development of roots and shoots throughout the observation period. In this case, as a function of time (between day 3 and day 12), root length increased from 5 to 72 cm when using hydropriming technology and from 21 to 96 cm when using POE-gel as a nanopriming agent. A similar behavior was observed for shoot length, which increased from 11 to 21 cm (hydropriming) and from 23 to 40 cm (POE-gel), as shown in Figure 2. On the 12th day, the root and shoot lengths presented average improvements of 33% and 90%, respectively, compared to the control group. Hence, these assays revealed (i) an accelerated germination process in the seeds primed with POE-gel and (ii) the formation of healthier seedlings relative to the control group. These findings introduce a novel application of the amine-epoxide gel in the field of agricultural science.

The remarkable germination acceleration and vigorous growth observed for the seeds subjected to POE-gel priming could be attributed to the distinctive characteristics of the system. Specifically, the entangled polymer chains formed swollen nanosized networks composed of hydrophilic POE, which facilitated molecular diffusion, thereby enhancing water uptake by the cucumber seeds. This phenomenon was consistent with previous work showing the potential of carbon nanotubes (CNTs) to improve plant cell growth,^60^ where the effect of the CNTs on cell growth was primarily ascribed to the formation of new channels that amplified the permeation and capillary transport of water. Numerous reports have shown the key role of facilitated water diffusion in promoting optimal plant growth,^61−65^ as well as the effect of nanoparticle treatment in enhancing seedling growth.^66,67^ Nanoparticle treatment has also been found to induce embryo activation, leading to the inadvertent production of enzymes that increase the preparedness of seeds for germination.^68^ The observed improvements, including accelerated germination and enhanced root and shoot length in cucumber plants, suggest a mechanism linked to the ability of POE nanoparticles to efficiently permeate cellular barriers such as cell walls and seed coats. This permeability likely facilitates sustained water uptake by the seed following the priming process, thereby promoting early and vigorous seedling development. Table S1 summarizes the effects of some nanomaterials in seed priming that can boost the development of seeds and improve seedling vigor and stress tolerance, leading to a more efficient agricultural process.

Gutiérrez et al.^69^ brought attention to the ongoing development of nanomaterial-based technologies for precision delivery of macro- and micronutrients to support optimal plant growth, considering essential elements, such as iron (Fe), nitrogen (N), molybdenum (Mo), magnesium (Mg), and others. Given these advances and the beneficial effects of the amine-epoxide system on cucumber seed germination (Figure 2), Fe^3+^ was incorporated as a micronutrient in the POE-gel at concentrations ranging from 10 to 100 mg·L^–1^ to investigate its effect on seed germination. Aqueous solutions consisting of water and the Fe^3+^ source at the same concentrations were employed as controls. The systems were denoted as POE-gel-Fe_*x*_ and control-Fe_*x*_, where x is the concentration of Fe^3+^ in the polymeric gel and aqueous solution. This experimental design was used to systematically assess the influence of the POE-gel-Fe_*x*_ systems on the progression of cucumber seed germination, shedding light on the potential benefits of introducing Fe^3+^ as a micronutrient within the POE-gel. The choice of Fe^3+^ was based on its crucial role as a micronutrient in biochemical processes within plant tissues. Iron is involved in critical functions, such as chlorophyll biosynthesis, mitochondrial respiration, and nitrogen fixation. It is also an essential constituent in enzymes, including catalase, ferredoxin, and peroxidase.^70^

Figure S6 and Table S2 show the results for the lengths of roots and shoots of germinated cucumber seeds primed with the POE-gel-Fe and the control-Fe solution, using three different Fe^3+^ concentrations (10, 50, and 100 mg·L^–1^). The germination process was monitored over a period of 12 days. Regardless of the Fe^3+^ concentration, the POE-gel-Fe treatments consistently exhibited comparable or enhanced germination evolution (considering both root and shoot lengths) compared to the control-Fe solutions. The use of Fe^3+^ at concentrations of 10 and 50 mg·L^–1^ resulted in no significant differences in root length between the control-Fe and POE-gel-Fe treatments (Figure S6a,b). However, the seeds treated with the POE-gel-Fe containing 100 mg·L^–1^ of Fe^3+^ presented a substantial positive effect on root length, exhibiting a threefold increase in size compared to the control-Fe treatment at the same concentration (Figure S6c). In addition, an increase in the Fe concentration in the control solution had a negative effect on root growth, with a decrease in length suggesting metal ion toxicity due to excessive exposure to 100 mg·L^–1^ of Fe^3+^ (Figure S6a–c, cyan-colored bars). Conversely, the results for shoot length showed that treatment with the POE-gel-Fe led to only slight increases at the different iron concentrations (Figure S6d–f). The outcomes obtained on the 12th day, using the POE-gel-Fe and control-Fe solutions at iron ion concentrations ranging from 10 to 100 mg·L^–1^, are discussed in detail below.

**Figure 3:**
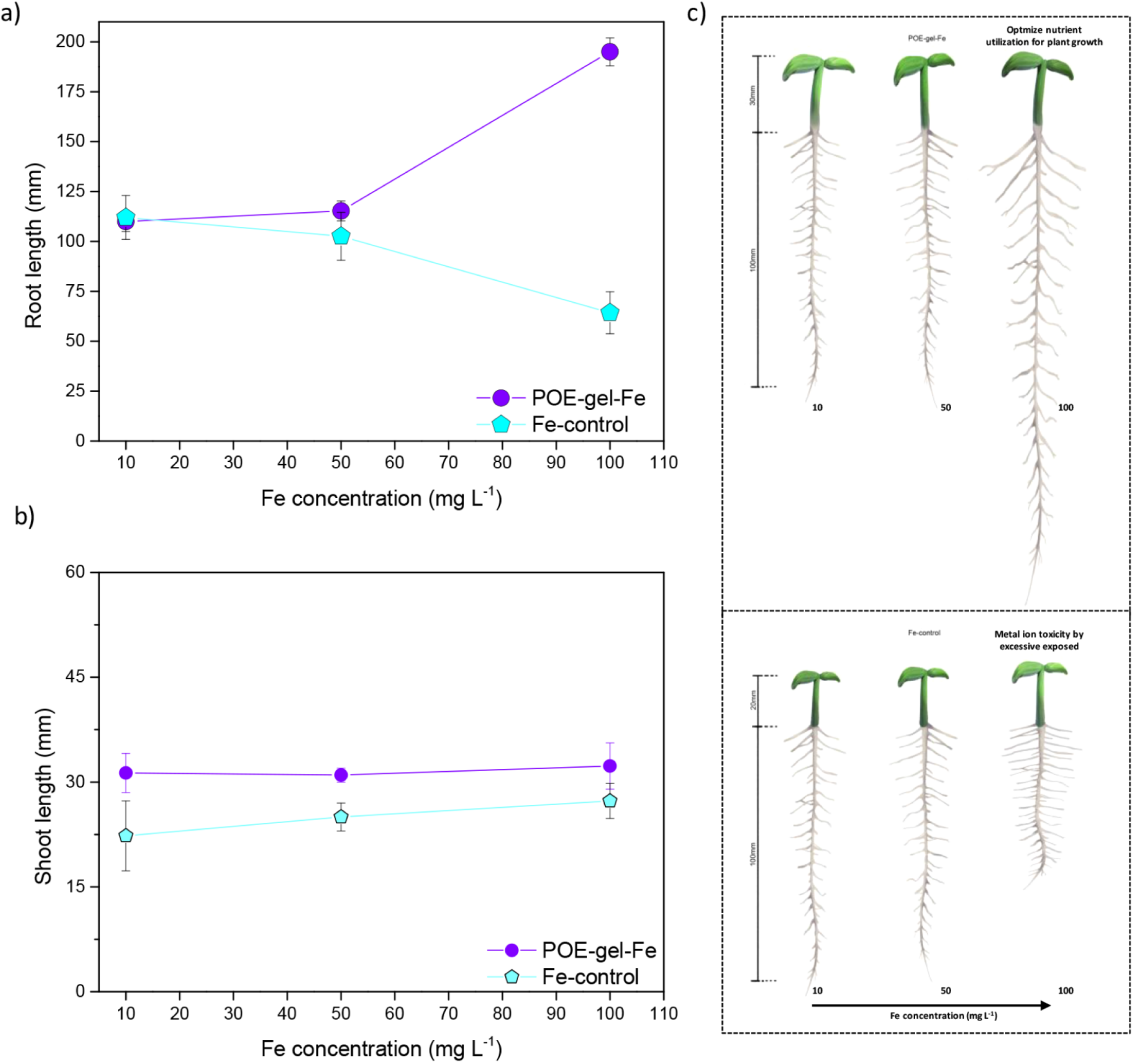
Mean values for the lengths of (a) roots and (b) shoots on the 12th day, comparing the loaded POE-gel-Fe and control-Fe seed priming treatments, using Fe^3+^ concentrations from 10 to 100 mg·L^–1^; (c) cartoon illustration of the plant size (roots and shoots lengths after day 12) by using loaded-POE-gel-Fe (positive effect) and control-Fe solutions (negative effect) as a function of Fe^3+^ concentrations.

A comparison was made of the effects of the loaded-POE-gel-Fe and control-Fe solution treatments, at the same iron concentrations, on the average root and shoot lengths obtained on day 12 after seed sowing (Figure 3). Distinct root growth behaviors were observed in response to the different Fe^3+^ concentrations after seed priming with loaded-POE-gel-Fe and control-Fe solution. For the control-Fe solutions, an increase in Fe^3+^ concentration (from 10 to 100 mg·L^–1^) appeared to lead to inhibition of root growth (Figure 3a). This could be attributed to the uptake of free and readily available iron ions, with toxicity effects at Fe^3+^ concentrations exceeding 50 mg·L^–1^, which hindered root development. Previous studies have demonstrated the adverse effects of high iron oxide nanoparticle concentrations (ranging from 30 to 150 mg·L^–1^) on seed germination and the development of roots and shoots, suggesting phytotoxicity.^46,71^ The present results were consistent with earlier findings, revealing that direct contact with Fe species in solution (≥50 mg·L^–1^) suppressed root development.

In contrast, the results for the seeds treated (nanoprimed) with loaded-POE-gel containing Fe^3+^ ions strongly suggested the remarkable effectiveness of the nanogels as micronutrient carriers to enhance root growth. The notable improvement in root growth using loaded-POE-gel-Fe, compared to the control-Fe solution (both with Fe^3+^ at 100 mg·L^–1^), suggested positive impacts of the nanogels by a combination of the following factors: (1) the POE-gel-Fe easily penetrated through the seed coating, acting as a nutrient carrier; (2) diffusion of the POE-gel particles facilitated the introduction of Fe^3+^ species into the seed; and (3) the release of Fe^3+^ was predominantly controlled by the structured hydrophilic amine-epoxide gel, resulting in a stimulating effect on root growth without inducing toxicity, even at the highest Fe^3+^ concentration (100 mg·L^–1^). Roots are essential for the survival and development of plants, performing critical functions such as anchoring the plant to the substrate, providing mechanical support, and absorbing water and nutrients. This work highlights the class of nanogels based on amine-epoxide as sustainable systems to enhance the evolution of plant root systems, opening a set of possibilities for advances in novel technologies for agricultural purposes. Introducing these nanogels (loaded and unloaded-POE-gel) as seed priming agents, no negative effects (toxicity) were observed during the seed assays. For shoot length, no significant alterations in growth were observed during the 12-day germination assays when different Fe^3+^ concentrations were used in both the control-Fe and POE-gel-Fe treatments (Figure 3b). Figure 3c showed a cartoon illustration of the effect of Fe^3+^ amount after seed priming with Fe-control solutions (negative effect) and loaded-POE-gel-Fe (positive effect) as a function of metal ion concentration (from 10 to 100 mg·L^–1^), respectively. These findings underscore the potential of POE-based nanogels as carriers to optimize nutrient utilization for enhancing cucumber seedling growth. A cost estimation for producing the POE-gel under the synthesis conditions described in this study was conducted. Based on the laboratory-scale prices of identifiable components, the estimated total cost-calculated as the sum of individual cost elements-was approximately $0.16 per kilogram of seeds (see Table S3 for details). This amount of POE-gel aqueous solution is sufficient to coat approximately 32,000 cucumber seeds, based on the average weight of one kilogram of seeds. Given its low production cost, this water-based POE-gel formulation presents significant potential for future application as a seed priming technology. Furthermore, this study highlights promising opportunities for the use of nanogels in agricultural applications.

**Figure 4:**
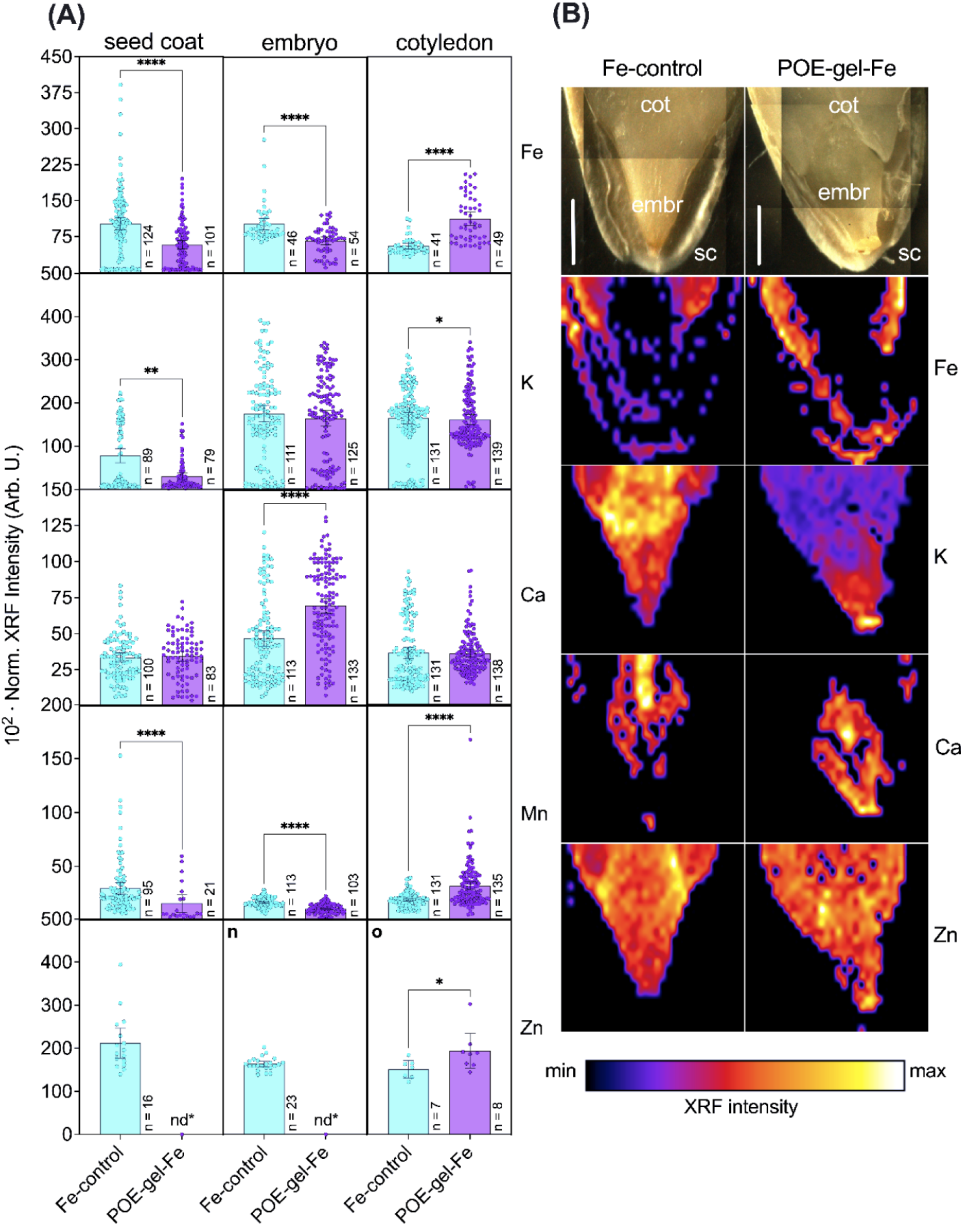
(A) Microprobe XRF analysis of cross sections of different tissues (seed coat, embryo, and cotyledon) of cucumber seeds primed with either the positive control (Fe solution) or the POE-gel-Fe. The Fe, K, Ca, Mn, and Zn intensities were recorded by using line scanning. The bars show the mean and standard error for several measurements recorded using three independent biological replicates. The *n* values are indicated at the base of the bars. The data were compared using the Mann–Whitney test at a 95% confidence level (*p* < 0.05). (B) Confirmation of the patterns by two-dimensional mapping. The intensities for manganese were below the instrumental limit of detection for the maps. Sc: seed coat; embr: embryo; cot: cotyledon. Scale: 1 mm.

The influence of loaded POE-gel-Fe nanopriming on seed germination and development was further investigated by analyzing the spatial distributions of elements in cucumber seeds using microprobe X-ray fluorescence spectroscopy (μ-XRF). For this purpose, seeds primed with the control-Fe solution and the POE-gel-Fe gel, at an Fe^3+^ concentration of 100 mg·L^–1^, were chosen, as this had been shown to cause a substantial enhancement of root growth. Figure 4A shows the results of XRF analysis of Fe, K, Ca, Mn, and Zn in cross-sections of the seed coats, embryos, and cotyledons of *C. sativus* seeds primed with the control-Fe solution and the POE-gel-Fe. Interestingly, a higher Fe intensity was found in the cotyledons of the seeds primed with POE-gel-Fe, with a similar trend observed for the intensities of K, Mn, and Zn. This phenomenon can be attributed to the presence of functional groups within the polymeric network of the nanogel, such as amine, glycols, and ether-type oxygen (from the hydrophilic POE backbone), which impact its capacity to extract water and carry nutrients from the surroundings. In contrast, higher Fe intensities were found in the seed coats and embryos of seeds exposed to the positive control. These patterns were confirmed by two-dimensional XRF mapping (Figure 4B). The XRF scanning for each biological replicate is detailed in Figures S3 and S4. When a solution containing Fe^3+^ ions was added to the amine-epoxide networks, these ions could diffuse into the polymeric structure of the nanogel, suggesting the formation of a hybrid system based on amine-epoxide-Fe^3+^ complexes, where the amine groups of the poly(etheramine)-POE or the hydroxyl groups generated during the epoxide ring-opening process were able to bind ion species. Kras et al.^72^ demonstrated the coordination of Fe(III) with amide and hydroxypropyl groups, forming promising complexes for potential applications as magnetic resonance imaging (MRI) probes.

As mentioned above, Fe^3+^ is involved in key biochemical processes, including the photosynthesis electron transfer system.^73^ In most seeds, Fe^3+^ is located within the vacuoles of the embryo and cotyledon cells. Its VIT1 transporter-regulated movement throughout the tissues is reported to be associated with germination.^74^ Iron deficiency-related detrimental effects have been described in several species, including chickpea,^75^ wheat,^76^ and soybean.^77^

Iron-based seed treatment has also been shown to have beneficial effects in other species, such as spinach,^78^ dill,^79^ sorghum,^80^ rice,^81^ and beans.^82^ Therefore, the findings suggest that the greater effectiveness of the POE-based nanogel in increasing the Fe^3+^ concentration in the cotyledons of primed *C. sativus* seeds (Figure 4A,B) is closely linked to its synergistic effect in enhancing germination and seedling development, involving the controlled and sustained release of the embedded Fe^3+^ ions from the nanogels during germination.

Interestingly, the results also revealed higher intensities for K, Mn, and Zn in the cotyledon tissues of the seeds primed with the POE-Fe-gel. It is well known that K^+^ is a major regulator of plant homeostasis,^83^ while Mn^2+^ and Zn^2+^ act as cofactors in many physiological processes in plants.^84−86^ The observed pattern indicated a synergy between Fe^3+^ accumulation and the contents of K^+^, Mn^2+^, and Zn^2+^, which could have contributed to *C. sativus* germination and development. Despite the sensitivity of seeds to external agents in nanoparticle-based treatments, both the unloaded and iron-loaded POE-nanogels demonstrated nontoxicity post-treatment, highlighting their safety and biocompatibility as promising systems for agronomic applications.

In-depth evaluation of the safety and prospective agricultural applicability of the amine-epoxide POE-gel was performed using *in vivo* assays with zebrafish (*D. rerio*), an established biological model employed to determine the biosafety profiles of diverse natural or synthetic materials, including nanoparticles, pharmaceuticals, and polymers.^87,88^ The aims of this assessment were to elucidate potential environmental implications of the presence of the amine-epoxide gel in aquatic ecosystems and to obtain information concerning possible adverse effects on human health. A benefit of using zebrafish as a model organism is that it has genetic similarity to humans.^89^

**Figure 5:**
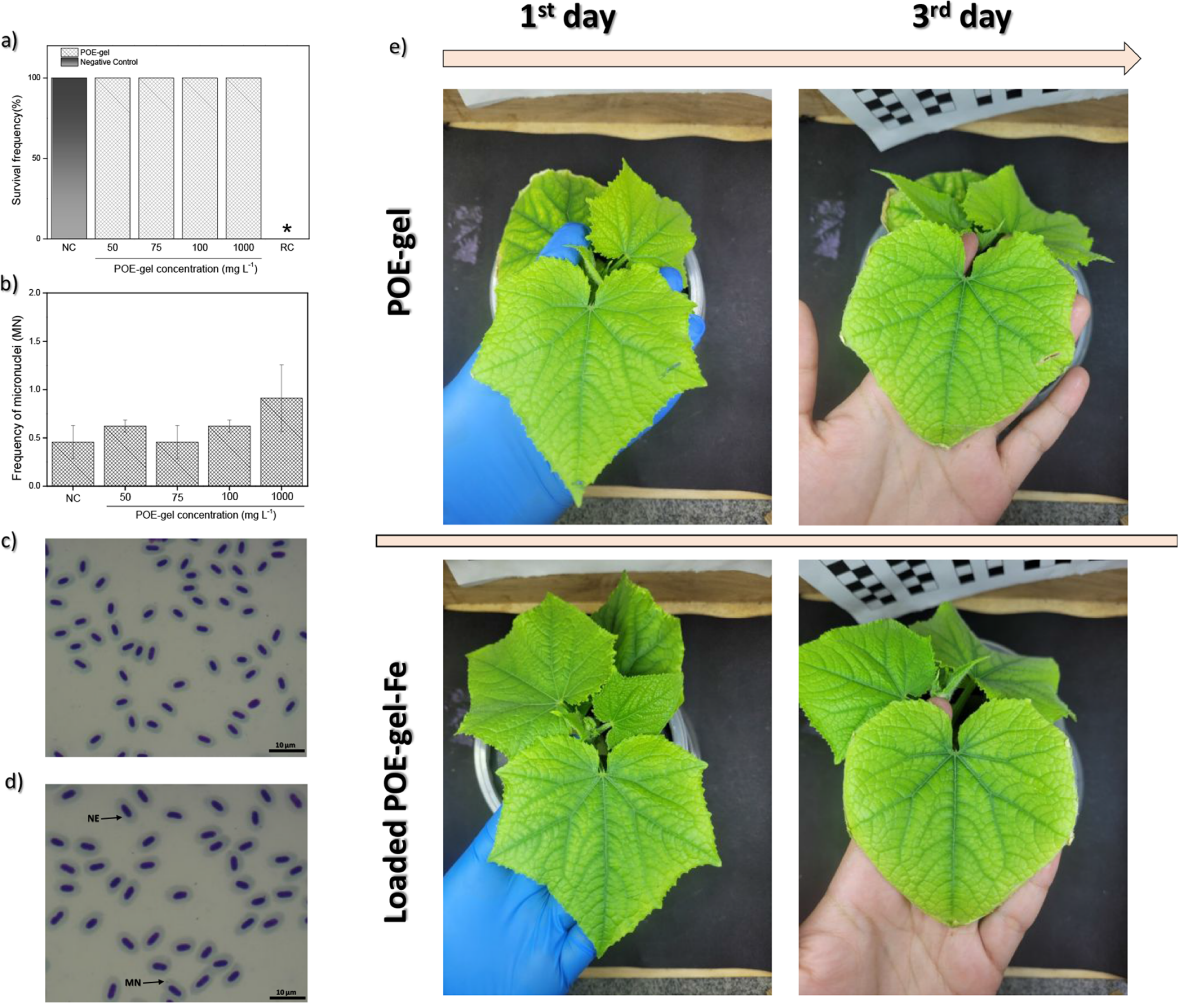
(a) Survival frequency of zebrafish after 96 h of exposure to different concentrations of POE-gel (from 50 to 1000 mg·L^–1^—aqueous solution system with spherical particles corresponding to the TEM image from Figure 1c), together with the water negative control (NC) and the reference control (RC, standardized red propolis extract at a concentration of 25 mg·L^–1^). Seven fish per treatment group were used. (b) Frequency of micronuclei (MNi) for the POE-gel and NC groups. Blood smears of adult zebrafish after 96 h of exposure to (c) the water NC group and (d) POE-gel at 1000 mg·L^–1^. The black bar indicates 10 μm. NE: normal erythrocyte; MN: erythrocyte with micronucleus. *Significantly different from the negative control group (*p* < 0.05). (e) Photographs illustrating the effects of loaded and unloaded POE-gel on cucumber surface foliage—72 h after application.

Adult zebrafish were exposed to 50, 75, 100, and 1000 mg·L^–1^ of POE-gel for 96 h. No mortality was detected in the water negative control (NC), and no significant difference was observed among the different POE-gel treatment groups (Figure 5a). The occurrence of micronuclei (MNi) in the erythrocytes of the fish exposed to different concentrations of POE-gel did not differ statistically from that of the NC (Figure 5b). A comparison of blood smears for the adult zebrafish belonging to the NC and POE-gel (1000 mg·L^–1^) groups is provided in Figure 5c,d. Likewise, exposure of the zebrafish to the amine-polyether-epoxide-loaded and unloaded POE-gel did not result in significant nuclear abnormalities compared to the NC group. For both the NC and the POE-gel exposures, only the occurrence of micronuclei was found. These results were indicative of a high degree of *in vivo* biocompatibility, with no acute toxicity of the POE-gel in adult zebrafish.

The use of polymer-based nanomaterials and nanotechnology has been demonstrated to have the potential to enhance the efficiency of current agricultural practices by improving the delivery of agrochemicals to plants, consequently reducing the requirement for fertilizers and pesticides. However, the application of polymers for nanoenabled seedling enhancement has received less attention. The findings of this work support the viability of employing POE gel as an eco-friendly priming agent and as a carrier for increasing the delivery of essential nutrients to seed tissues. This system could potentially be used for the protection of seeds, emerged seedlings, and plants from diseases and pests, as well as in the provision of soil adjuvants, growth regulators, and polymer coatings for the long-term storage of seeds.

A preliminary assay was performed with the application of the POE-gel to the surfaces of cucumber leaves (Figure 5e). Entire leaves were coated with POE-gel (unloaded and loaded with Fe, 100 mg·L^–1^) using a brush, followed by evaluation of the responses after 3 days. No signs of phytotoxicity were observed, further evidencing the biocompatibility of the nanogel and supporting its potential future use as a foliar nutrient delivery system for plants.

## Conclusions

A liquid-phase process was successfully used to synthesize an amine-epoxide based on polyoxyethylene (POE chains), offering applications in agriculture as seed priming technology. This research introduces pioneering concepts, particularly for seed germination enhancement, filling a gap in the literature regarding the use of polymeric gels to provide beneficial effects by coating seed surfaces. An important finding was that the use of the POE gel as a seed priming agent did not cause any toxicity during the germination process of *C. sativus* seeds. The potential of this polymeric system is further supported by its simple synthesis and the ability to incorporate essential plant nutrients such as iron ions. Notably, the ability of the POE gel to facilitate the internalization of essential micronutrients (Fe, K, Mn, and Zn) without exhibiting toxic effects is of high significance, addressing both agronomic efficiency and ecological safety concerns. This methodology has the potential to be expanded to include a variety of macro- and micronutrients, opening numerous opportunities to improve crop yields. Future studies will focus on further investigation of ionome dynamics during seed germination, utilizing a panel of plant species including soybean and maize. Additionally, an investigation will be made into the effects of this gel on foliar ion absorption, thereby contributing to a comprehensive understanding of the potential applications of the POE gel system in diverse agricultural contexts.

## Supporting Information Available

Details of the samples and strategy employed for μ-XRF mapping-line scanning, additional characterization ^1^H NMR, FTIR, seed development bar chart, and Tables with nanomaterials used as seed prime technology, values of root and shoot length and a detailed estimative cost (PDF)
