# Supplementary material for: Amine-Polyether-Epoxide Nanoplatform-Driven Seed Germination, Plant Growth, and Nutrient Uptake for Sustainable Agriculture
Source: ACS Omega. 2025 Mar 20;10(12):12453–66. doi: 10.1021/acsomega.4c11661 (PMC11966252; doi:10.1021/acsomega.4c11661)
Supplement: Supplementary file 1 — ao4c11661_si_001.pdf [file ao4c11661_si_001.pdf]

Supporting Information

Amine-Polyether-Epoxide Nanoplatform-Driven Seed Germination, Plant Growth, and Nutrient Uptake for Sustainable Agriculture.

Bruno A. Fico [1], Heber E. Andrada [1], Felipe B. Alves [1], Enzo E. da Silva[1], Julia S. Reinaldi [1], Denise C. Tavares [1], Iara S. Squarisi [1], Laura G. Nuevo [2], Gabriel Sgarbiero Montanha [2,†], Hudson W. P. de Carvalho [2, ‡], Fabián Vaca Chávez [3,§], Eduardo F. Molina [1]*

[1] Universidade de Franca, Av. Dr. Armando Salles Oliveira 201, Franca, SP, 14404-600, Brazil

[2] Grupo de Estudo em Fertilizantes Especiais e Nutrição, Centro de Energia Nuclear na Agricultura, Universidade de São Paulo, Av.Centerário 303, Piracicaba, SP, 13400-970, Brazil

[3] Universidad Nacional de Córdoba, Facultad de Matemática, Atronomia, Física y Computación, Córdoba, Argentina.

*Corresponding author e-mail: eduardo.molina@unifran.edu.br

[†] Dipartimento di Biologia e Biotecnologie Charles Darwin, Sapienza Università degli Studi di Roma ‘La Sapienza’, Via dei Sardi 70, Roma, RM, 00185, Italy
[‡] Global Critical Zone Science, Mohammed VI Polytechnic University, Ben Guerir 43150, Morocco
[§] CONICET, Instituto de Física Enrique Gaviola (IFEG), Córdoba, Argentina

Supporting Figures

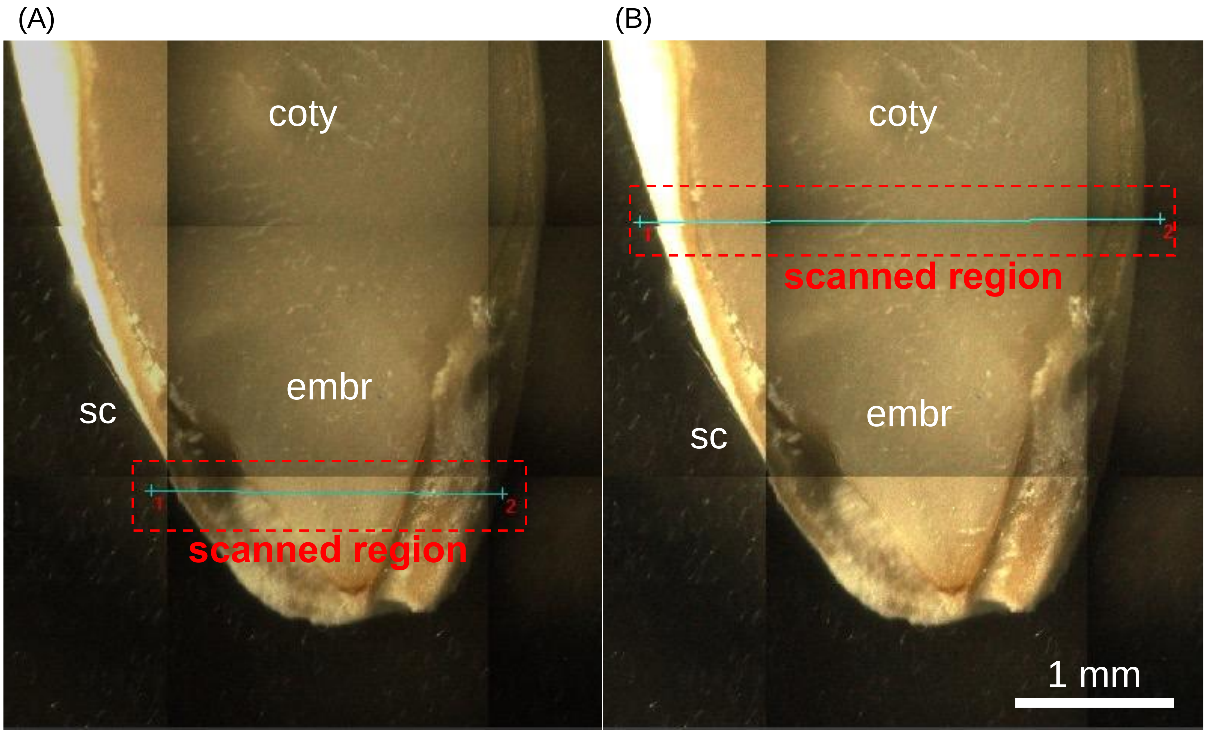

Figure S1. Details of the samples and experimental strategy employed for μ-XRF assessment of ionome distribution in cucumber seeds.

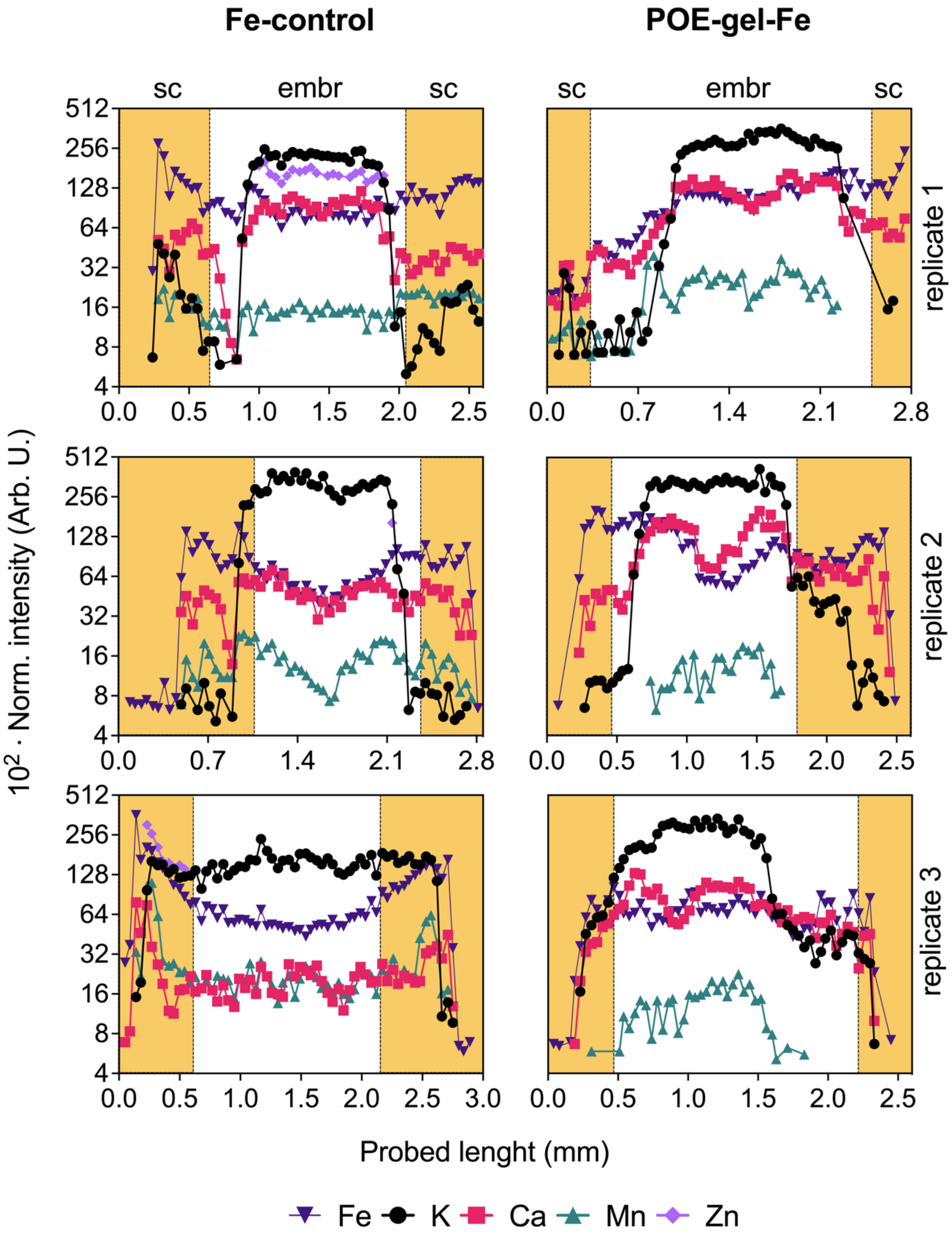

Figure S2. Normalized µ-XRF line scanning of Fe, K, Ca, Mn, and Zn distribution in cucumber seed cross-section primed with positive control (Fe solution) or the POE-gel-Fe solution. The data within the yellow boxes regards to the values recorded on the seed coat, whereas the white box indicates those recorded in the embryo tissues. The analyses were carried out using three independent biological replicates. Only the values above the instrumental limit of detection are presented.

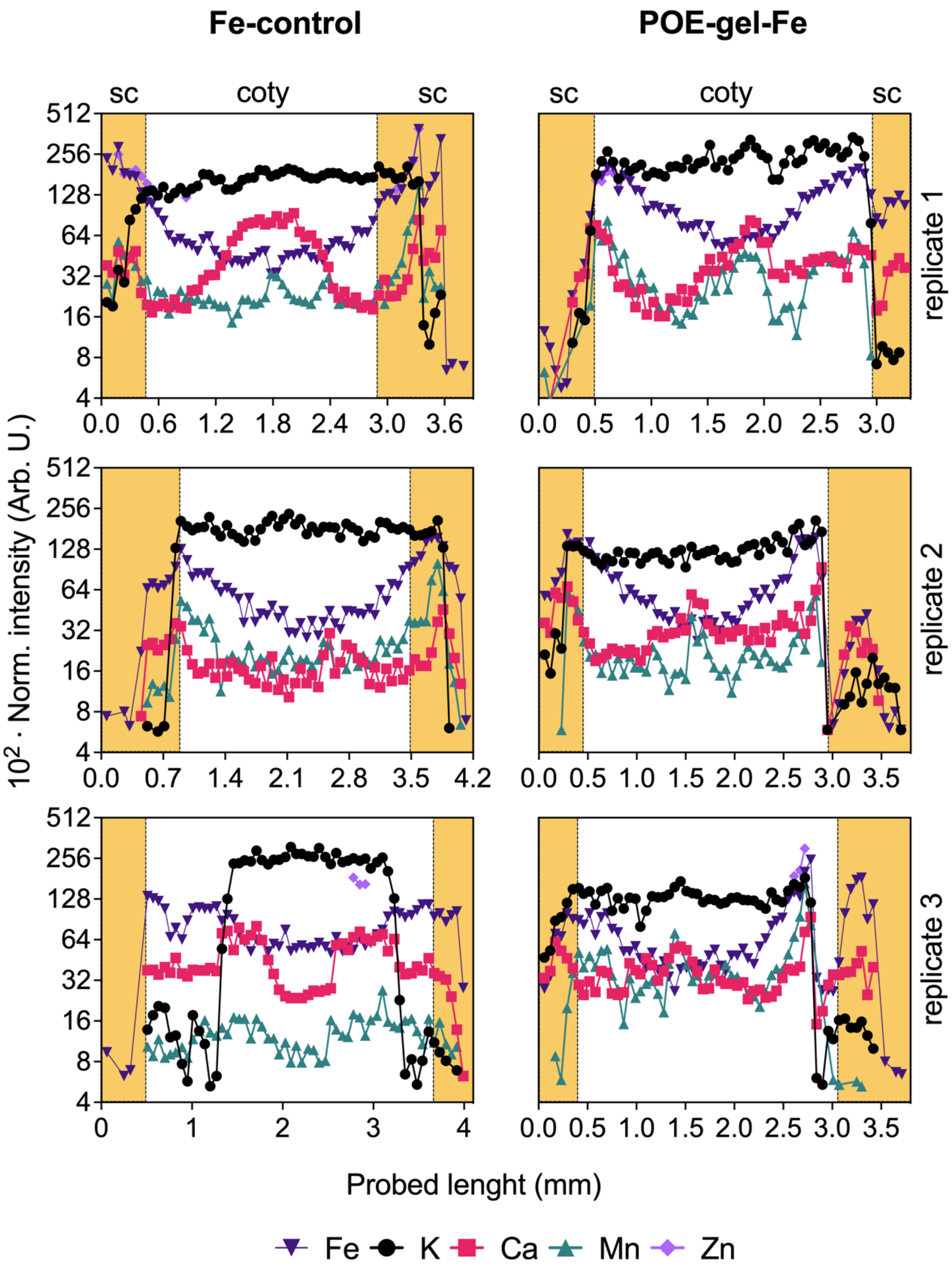

Figure S3. Normalized µ-XRF line scanning of Fe, K, Ca, Mn, and Zn distribution in cucumber seed cross-section primed with positive control (Fe solution) or the POE-gel-Fe gel solution. The data within the yellow boxes regards to the values recorded on the seed coat, whereas the white box indicates those recorded in the cotyledon tissues. The analyses were carried out using three independent biological replicates. Only the values above the instrumental limit of detection are presented.

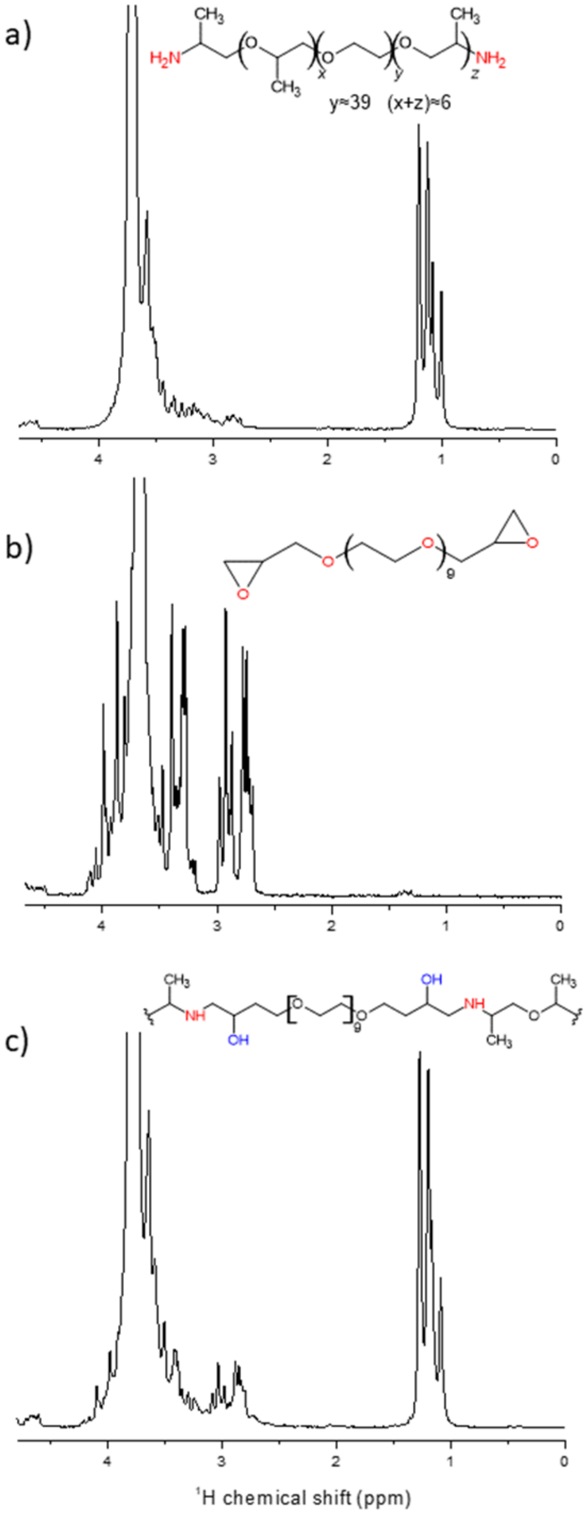

Figure S4. Representative ^{1}H NMR spectrum of (a) Jeffamine ED2003, (b) DPEG epoxide and (c) POE-gel. All the ^{1}H-NMR measurements were performed on aqueous solutions.

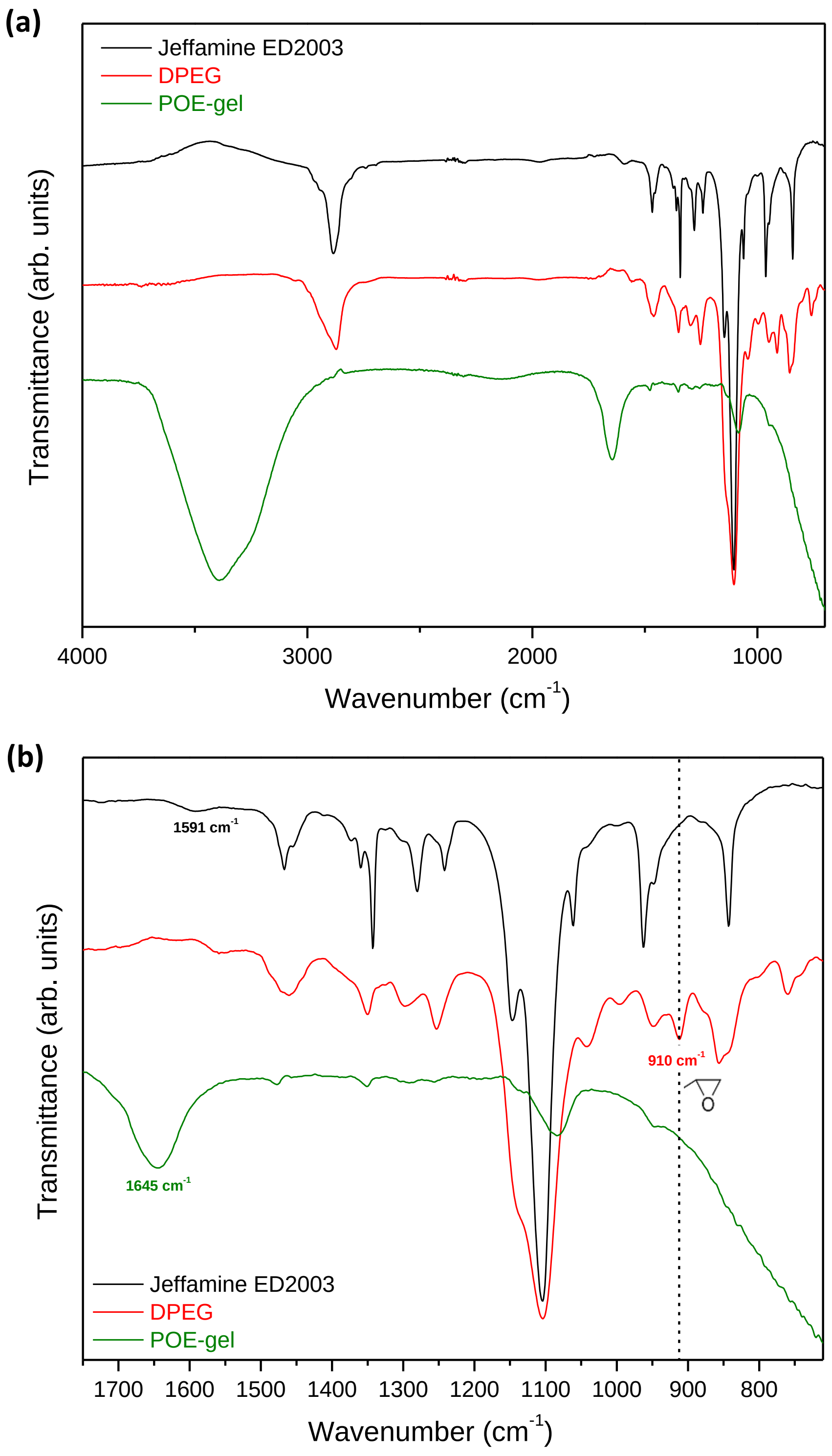

Figure S5. FTIR spectra in the region from (a) 700 – 4000 cm^{-1} and from (b) 700 – 1750 cm^{-1} for Jeffamine ED-2003 (black line), DPEG (red line), and POE-gel (red line).

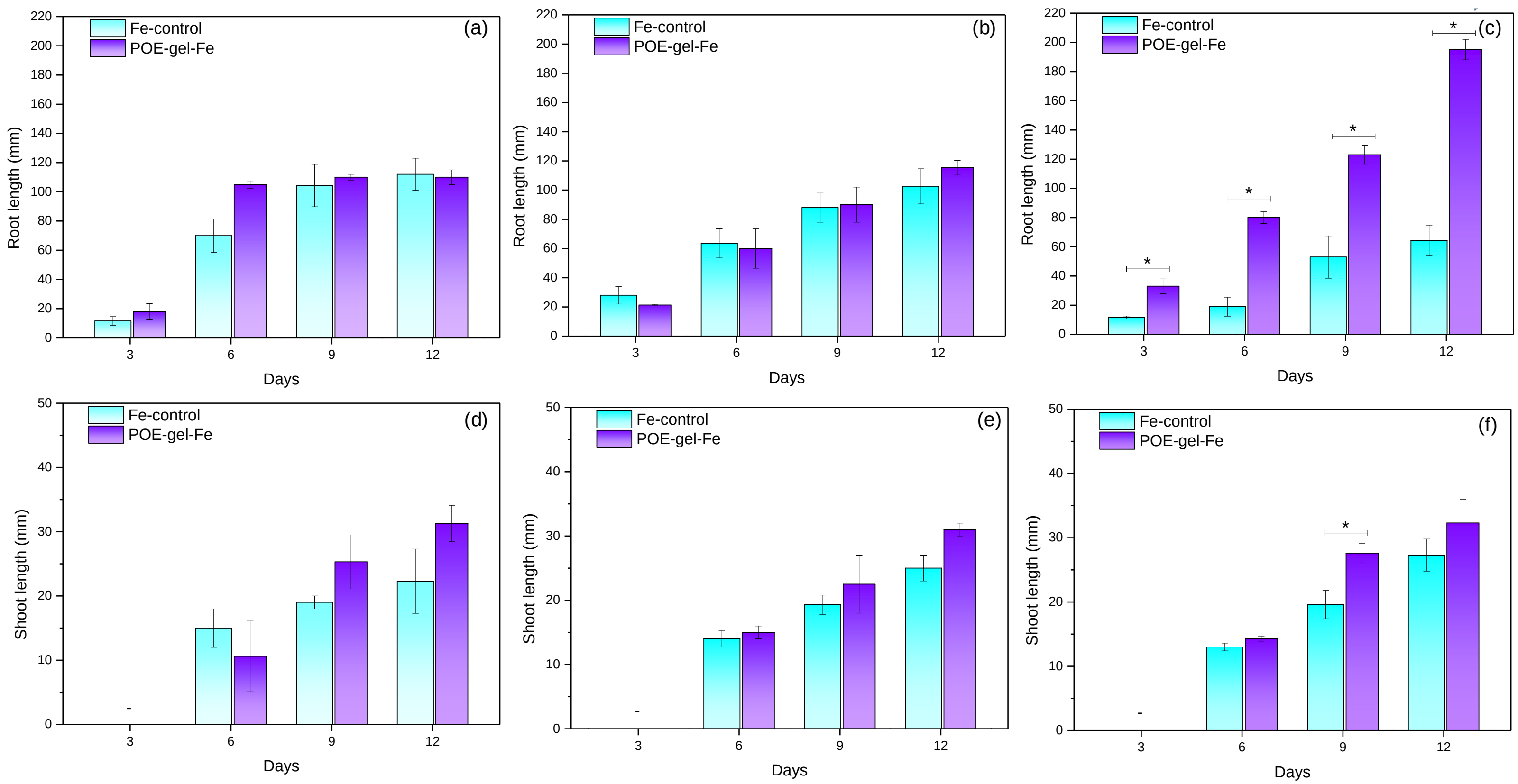

Figure S6. Influence of Fe^{3+} concentration on initial seedling growth during 12 days after sowing, for treatments using the control-Fe solutions and the POE-gel-Fe gels. Evolution of (a-c) root length and (d-f) shoot length, for treatments using 10 mg L^{-1} (a,d), 50 mg L^{-1} (b,e), and 100 mg L^{-1} (c,f) of Fe^{3+}. Statistical significance was determined using one-way ANOVA with multiple comparisons (*$p < 0.05$). Unless indicated, there was no statistical difference. (-) indicate initial germination process with radicals emerging from the seeds.

Table S1. Summary of reported nanomaterials used for seed treatment

Nanomaterial used for seed treatment	Crop	Amount	Seed steril -method	Main effect	Ref
FeS_2	*Cicer arietinum L.*	20 - 100 mg L^{-1}	10% hypochlorite solution	increased plant biomass	[1]
Chitosan nanoparticles	*Triticum aestivum L.*	1 - 100 mg L^{-1}	75% ethanol for 5 min	seed germination and seedling growth	[2]
Multi-walled carbon nanotubes	*Ricinus communis L.*	2 - 500 mg L^{-1}		Increase in biomass and number of roots.	[3]
Graphene oxide	*Cucumis melo L.*	250 – 1000 mg L^{-1}	5% sodium hypochlorite solution for 2 min	Resistance to saline stress	[4]
Carbon-based nanoparticle	*Momordica charantia*			possibility of improving crop yield and quality	[5]
Fullerol	*Triticum aestivum L.*	0 – 300 mg L^{-1}		improving seed germination, seedling growth and antioxidant enzyme activities	[6]
Ag nanoparticles	*Triticum aestivum L.*	0 – 50 mg L^{-1}	10% sodium hypochlorite solution for 10 min	Promotes growth, increased biomass and root elongation.	[7]
Chitosan	*Zea mays L.*	Solution at 1% and 2% (wt./v)	70% ethanol and 0.1% mercuric chloride	Improved osmotic stress resistance	[8]
Polyethylene glycol -PEG	*Triticum aestivum L.*	PEG solution + Water potential from −0.3 to −1.8 MPa		higher germination rate	[9]
POE-gel	*Cucumis sativa*	10-100 mg L^{-1}	2% sodium hypochlorite for 15 min	increased root growth and stem development	this work

Table S2. Summary of the root and shoot length values (mm) during seedling growth as a function of time (days) for control-Fe solutions and POE-gel-Fe containing 10, 50 and 100 mg L^{-1} of Fe^{3+}. The values are represent as means for all measures (data collect using 15 seeds per treatment). * statistical significance by employing one-way ANOVA *$p < 0.05$ and NS, nonsignificant

	Germination evolution parameters (days)							
Seed treatment	Root Length (mm)				Shoot Length (mm)			
	3rd	6th	9th	12th	3rd	6th	9th	12th
Fe-control-10	11.6 NS	70.1 NS	104.3 NS	112.0 NS	-	15.0 NS	19.2 NS	22.3 NS
POE-gel-Fe-10	18.1 NS	105.0 NS	110.2 NS	110.1 NS	-	10.6 NS	25.3 NS	31.3 NS
Fe-control-50	28.0 NS	63.6 NS	88.3 NS	102.6 NS	-	14.0 NS	19.3 NS	25.1 NS
POE-gel-Fe-50	21.3 NS	60.2 NS	90.0 NS	115.3 NS	-	15.0 NS	22.5 NS	31.2 NS
Fe-control-100	11.5*	19.0*	53.0*	64.3*	-	13.0 NS	19.6*	27.3 NS
POE-gel-Fe-100	33.0*	80.4*	123.0*	195.1*	-	14.3 NS	27.6*	32.3 NS

Table S3. Estimated cost to produce POE-gel based on the amount of raw materials used during the syntesis procedure. The price of raw materials was based on Aldrich values of the reagents.

Raw material	***Amount used in this work***	***Estimated Cost*** (US$)
Jeffamine ED-2000	800 mg	0.350
Epoxide DPEG	200 mg	0.068
**Total**		**0.418**

*the estimated cost (approximately forth cents) with the amount of raw materials cited above it is possible to produce a POE-gel solution at 0.5 %wt used to coat ~2.64 kg of seeds. This means approximately 85.000 seeds treated - number based on weight of a single cucumber seed used in this work.

References

(1) Srivastava, G.; Das, A.; Kusurkar, T. S.; Roy, M.; Airan, S.; Sharma, R. K.; Singh, S. K.; Sarkar, S.; Das, M. Iron Pyrite, a Potential Photovoltaic Material, Increases Plant Biomass upon Seed Pretreatment. *Materials Express* 2014, *4* (1), 23–31.

(2) Li, R.; He, J.; Xie, H.; Wang, W.; Bose, S. K.; Sun, Y.; Hu, J.; Yin, H. Effects of Chitosan Nanoparticles on Seed Germination and Seedling Growth of Wheat (Triticum Aestivum L.). *Int J Biol Macromol* **2019**, *126*, 91–100.

(3) Fathi, Z.; Khavari Nejad, R. A.; Mahmoodzadeh, H.; Satari, T. N. Investigating of a Wide Range of Concentrations of Multi-Walled Carbon Nanotubes on Germination and Growth of Castor Seeds (Ricinus Communis L.). *J Plant Prot Res* **2017**, *57* (3), 228–236.

(4) Kaymak, H. Ç.; Sevim, M.; Metin, Ö. Graphene Oxide: A Promising Material for the Germination of Melon Seeds under Salinity Stress. *Turkish Journal of Agriculture and Forestry* **2022**, *46* (6), 863–874.

(5) Kole, C.; Kole, P.; Randunu, K. M.; Choudhary, P.; Podila, R.; Ke, P. C.; Rao, A. M.; Marcus, R. K. Nanobiotechnology Can Boost Crop Production and Quality: First Evidence from Increased Plant Biomass, Fruit Yield and Phytomedicine Content in Bitter Melon (Momordica Charantia). *BMC Biotechnol* **2013**, *13* (1), 1–10.

(6) Kong, H.; Meng, X.; Akram, N. A.; Zhu, F.; Hu, J.; Zhang, Z. Seed Priming with Fullerol Improves Seed Germination, Seedling Growth and Antioxidant Enzyme System of Two Winter Wheat Cultivars under Drought Stress. *Plants* **2023**, *12* (6), 1417.

(7) Kannaujia, R.; Srivastava, C. M.; Prasad, V.; Singh, B. N.; Pandey, V. Phyllanthus Emblica Fruit Extract Stabilized Biogenic Silver Nanoparticles as a Growth Promoter of Wheat Varieties by Reducing ROS Toxicity. *Plant Physiology and Biochemistry* **2019**, *142*, 460–471.

(8) Kakar, H. A.; Ullah, S.; Shah, W.; Ali, B.; Satti, S. Z.; Ullah, R.; Muhammad, Z.; Eldin, S. M.; Ali, I.; Alwahibi, M. S.; Elshikh, M. S.; Ercisli, S. Seed Priming Modulates Physiological and Agronomic Attributes of Maize (Zea Mays L.) under Induced Polyethylene Glycol Osmotic Stress. *ACS Omega* **2023**, *8* (25), 22788–22808.

(9) Lemmens, E.; Deleu, L. J.; De Brier, N.; De Man, W. L.; De Proft, M.; Prinsen, E.; Delcour, J. A. The Impact of Hydro-Priming and Osmo-Priming on Seedling Characteristics, Plant Hormone Concentrations, Activity of Selected Hydrolytic Enzymes, and Cell Wall and Phytate Hydrolysis in Sprouted Wheat (Triticum Aestivum L.). *ACS Omega* **2019**, *4* (26), 22089–22100.
